# The Constitution of Man

**Published:** 1889-10-19

**Authors:** Andrew Clark

**Affiliations:** President of the Royal College of Physicians


### The Constitution of Man.

By Sir Andrew Clark, Bart., M.D., F.R.S., President of the Royal College of Physicians.

*Being a Lecture delivered at Wribbenhall, Worcestershire.*

It is a very beautiful and very marvellous world in which it is our privilege to dwell, but, unhappily, not one in a hundred of us will stay to look at its beauty, or stay to think over its marvels. No; we have eyes to see, and we do not struggle to see; we have minds, and we do not strive to understand, and thus it is that in looking and thinking over these things we miss that expansion of thought, that serenity of heart, that elevation of the soul, which looking at these things should bring to everyone of us. Yes, it is a very beautiful world, this in which we dwell. Let us take some sunny day in June, and ascend one of the heights here just close to Bewdley, and look on the scene which is spread out before us. There are the beautiful fields, the fields of green grass and golden corn, and rich brown earth. There are the browsing cattle and innumerable multitudes of living things that find pleasure therein. There are the stately trees clapping their leafy hands and making songs thereby to the breeze as it blows in passing. There are the wooded hollows shrouded in mystic mists, and there is the silvery winding river pursuing its course to the distant sea and making music as it goes. There are the purple hills, and there over all is the great blue sky crowned with the summer sun radiating down its beams of light and heat upon a gladsome and responsive earth below. Here is the pleasant air laden with fragrance, instinct with life, and sounding with the songs of birds; and here, coming to us, as it were, from afar, is that sense of the Divine presence which fills the soul with awful and yet sweet joy. Surely it is beautiful. And above all, we see from this little world on which we dwell, there is the starry firmament with all its wonders.

Let us look out on some clear, dark, wintry night, and see the azure firmament studded with its galaxies of stars shining out of the darkness like lamps casting forth rays of silver. There, in these immeasurable fields, are systems upon systems of worlds, each complete in itself, each with its sun and its planets, each running its appointed course, and in such inextricable masses as almost staggers the mind to contemplate. These systems do not interfere with one another, but each is in such close relationship to the other that whenever anything happens to one, however far distant, it is felt as a kind of throb throughout the whole. We ask the telescope to help us when the eye ceases to be of use, and by its aid we see further into these illimitable fields of space, still further and further amongst the countless systems to worlds upon worlds, each moving in its own appointed course, and yet not one interfering with another. We ask imagination to help us where the telescope ceases, and imagination comes to help us till it staggers and fails at the sight, at the terrible immensity of that with which it is brought face to face. Yes, beautiful as is this world, wonderful as are these starry heavens, there is nothing in either of them more worthy of consideration, there is nothing so suggestive of the Divine Ruler of the whole as the man who is gazing at this world and at these stars.

What is the constitution of man? You will remember that in one of the first chapters of Genesis there occurs the statement that God said "Let us make man in our image and in our likeness." And so he did; and when we speak of God as the Trinity in unity, as Triune, so we have to speak of man. Man is a trinity in unity, he is triune. First of all, he possesses a body; secondly, a mind; and thirdly, a spirit. Now these parts are perfectly distinct one from the other, and the one cannot grow into the other, but altogether the three are man. Man has a body and life in common with plants. Life is possessed by a thing which comes from something like itself, which grows, develops, and fulfils certain functions or work in the world, and produces another thing like itself and dies. Plants have this qualification, and therefore man possesses body and life in common with plants. He possesses, secondly, mind—a mind in common with animals. We say a mind is present when anything which lives has a consciousness of its own existence and of the external world. A dog has a mind, for if you examine a dog you will see he is conscious of his own existence and of the world, and observes and in a certain degree regulates his acts, and therefore in some sense or another he has what we call a mind. But, lastly, man has a third element, a spirit, and this he possesses in common only with God. The spirit is the divine part of man which links him on one side with Nature and on the other side with God, in whose image he was made. Now this wonderful constitution with which man is furnished is like in a sense a well-ordered state. He has a body, the machinery, the executive wherewith to perform his work, he has a mind to deliberate—a council, to give orders; and, lastly, he has a spirit, supreme, the ruler of all, the spirit which is informed by the mind and which acts through the mind upon the body. This, then, is the constitution of man.

Now with regard to the body. It is in every respect subservient to the common laws of Nature—if you drop a stone it falls—and there is one thing about the laws of Nature—remember they are unalterable and inviolable. The whole machinery of man and the whole machinery of the universe work on laws unalterable and inviolable. And so also is the mind subject in all things to the ordinary laws of the physical constitution of man. But when we come to the spirit of man we find ourselves in a totally new region, a region of such mystery and marvel that the more we think of it the more are we impressed with the mysterious, and the more and more utterly hopeless do we become of any explanation. Let me introduce you a little more closely to the spirit. When you say my hand, my eye, my feelings, my will, what is this *my*? What do you mean by it? It is not your leg, your arm, or even your mind, for you may lose many faculties of the mind, but the mystery, "my," remains untouched. You may lop off all the limbs, but as long as you do not destroy life this *my*, this *I*, remains untouched exactly as before. That *I* is the spirit of man. What it is I cannot tell; it is there; it is eternal, ever present, a mystery, in his mind, in his body, in him, and through him, ruling him, and constituting, as I have said, the spirit of man. I have said that in speaking of the third part of man we go into the region of mystery. I have shown you where the mystery is, I have shown you it is present everywhere, and not one of you can tell what it is. It is the origin of what people call free will.

When we come to the spiritual part of man, the physical laws of nature cease to act, and for these laws there is substituted what is called a moral law. There comes in the notion of right and wrong, but where they come from I do not know at present, because this leads us to the point that laws are alterable and violable, and that this alterability constitutes in their infringement right and wrong. Here is only mystery, but here is freedom. It is a strange thing to which I have brought you face to face, this great mystery, this question of something totally new in the universe, moral laws which are alterable and violable by the will of man. The will of man. Do you mean to tell me there is such a thing as free will! Philosophers have proved there is no such thing as free will. Well, that does not prove to me conclusively that there is no free will, but it proves to me what I ask you to take seriously to heart, for it is a great saying that mere logic, mere intellectual processes are not a test, and are not a final measure of proof. I have no doubt that as a mere play of logic and reasoning it may be argumentatively sustained that there is no such thing as free will. But no serious man, of competent powers of reflection, will not and cannot doubt that besides these intellectual processes classed under the name of logic there are other guides to truth. There is feeling, sentiment, conscience—some of the finest discoveries in the world were not made by logic, but through the feelings, the instinct, the affections, the conscience, at which philosophers are apt to laugh. Let them laugh, for in their lives everyone of them act as though they believe their will is free.

I act on the assumption that the will is free, not because I shall prove it by strict logical reasoning, but, first of all, because I am conscious that I am free; secondly, because I can demonstrate in a moment that I am free; and, thirdly, because I am compelled to act in the practical business of life as if I am free; fourthly, because every other man is compelled to act as if free; and, lastly, because every argument which has been adduced against the possession of a free will is an argument which strikes at the cause of the action in man. The will is free. Then, if I am free, I am responsible. And to whom is this responsibility? I will give you that answer by-and-by. We are, then, in a region of freedom, of mystery, and of responsibility. We find ourselves called upon to act, and in every action it may be right or it may be wrong. How are we to know? First, there is what we call conscience, which is the illuminator of the third part of man, that is the spirit. The conscience illuminates the spirit in order that the will may be enabled to act according to that illuminator. But what illuminates the conscience? The only possible illuminator I can think of or imagine, or that I have ever heard of, is that Divine light which lighteth every man. Now, the constitution of man, as I have roughly sketched it, implies one thing more, the possession of what we may call causal power, the power of causing things. Everything in nature takes place through an unalterable eternal necessity, but it is only in the spirit of man we have the power of causing things. I do not pretend to say we can alter the law of nature, but we can take these laws and so combine them as to produce results exactly the same as if we were causing this from the laws themselves. The possession of this wonderful causal power makes man—I may say something which you may say is bordering on the irreverent, but it does not, for it is the sober truth, and it is this, that man at his highest and best is a model in miniature of the eternal God Himself. For in the highest development of the bodily life we have what is called health, for the mind what is called knowledge, and for the spirit what is called holiness.

What is health? I wish I could describe it. It is not a very common possession, although a most blessed one. It is that state in which we live without knowing that we have organs which are enabling us to live, that state in which we can perform all our functions and discharge all our duties without feeling ill at ease, and it is that state in which we not only enjoy ourselves, but also give joy to others. How is health to be got? Very simply, so simply that nobody—I won't say nobody—thinks it worth while to get it. Simple rules which if you obey, and if your fathers handed down to you a good constitution, will enable you to get good health; but, alas, fathers often eat the sour grapes, and the children's teeth are sadly set on edge. The laws of health are very simple—three meals a day, as little alcohol as possible, daily labour, warm clothing, sufficient sleep, sun, and air. These are very simple, but they are not always easier for being simple. It is very hard for us doctors to think that we live by the sins, the ignorance, and follies of mankind, but it is quite true, for with Nature there is no forgiveness of sins. If you disobey her laws when young she will wink at it, but the time of the payment of the penalty will come and she will exact the uttermost farthing. If people would only believe what is as true a truth as has ever been uttered, it is that labour is health. The highest life of any organ lies in the fullest discharge of the functions which belong to it.

With respect to knowledge—the life of the mind—I will say a word about the great mistake people are making nowadays. They talk about education, and say that it is the great age of education. Is it? I am not quite sure of that. I tell you it is undoubtedly the great age for cramming, and if merely storing the mind with facts, however varied, constitutes knowledge, then certainly it is the age of greatest knowledge. But if education is something different from that, something far higher, then I venture to say it is not a great age of knowledge. I take it education means two things; first, the development of the human faculties for the purpose of calling forth the use of knowledge, but mere cramming stints, starves, and keeps back. No, true education is the development of the faculties in fit relation to each other.

The condition of the spirit is holiness. What is holiness? It is the sustained effort to die to yourselves that you may live to God. But there is this peculiarity about it, you have not complete and unlimited freedom in dealing with holiness. There are two agents here needed. One is the desire, the struggle and striving of man for holiness, the other is the gift of God.

But you may say, "Is it really true?" You may believe me. I think so, or I would not have said it. Some of you sharp young bloods may say, "Oh, you are getting old, and do not perhaps know what men of science tell us nowadays. They tell us it is all bosh, all nonsense; they tell us that the dominion of the physical law—the law which is unalterable, inviolable, and eternal has invaded the whole dominion of man; that free will is a mere delusion; that vice and virtue, love and hatred, good and evil, are so many mere physical conditions of man; and that we cannot help ourselves because we are in the grip of that physical law which brings us we know not whence, and carries us we know not where. Then let us eat, drink, and be merry, for to-morrow we die." That is the materialistic, that is the scientific view? Well, do you like it? Ask yourselves if you hesitate, what is the history of every individual and the history of every nation which has thrived. Rome thrived—and I do not suppose it needs me to remind you that Rome tried to accept the materialistic view and acted upon it, and came to such a state of society which eventually led to things so awful that I dare not present in an assembly such as this. Do not everyone of you know somebody who is practically endeavouring to carry out life on these terms, and is it not true that everyone of them fails, and dies a moral death? No; to everyone of us the two modes of life are presented. No sooner do we come out of mere youth than we get into the presence of these two theories of life, one claiming everything for the body, and the other claiming everything for the spirit; one saying "I desire this, I desire that," and then when he seeks it and takes it and is enjoying it, there is the other life which says, "Thou art wrong to have it, it is wrong." Where does this voice come from? It is we know the voice of conscience. What right has conscience to speak? You know my answer. It is because the conscience is illuminated by God.

When every man and woman comes into the world these two theories of life are presented to them, and the whole of life is spent in this conflict between the spirit and the flesh to which the Apostle Paul so constantly alludes, and if we accept the view which indirectly I have presented to you it means that we shall be certainly on the side of the spirit. But to be on the side of the spirit I was going to say entails a most fearful sacrifice. Those who wish to be on the side of the spirit have to deny themselves every hour of the day and every day of their lives. It is a perpetual self-sacrifice, it is carrying out the best fulfilment of that strange and marvellous paradox which the Man Christ Jesus uttered whilst on earth, "He that seeketh his life shall lose it, and he that giveth it shall find it again." Are there no difficulties in accepting this terrible paradox? Yes, my friends, there are many difficulties, and we ought to thank God that there are. Why, what would you be without difficulties? If you had no bodily difficulties where would be your strength; without any intellectual difficulties, where knowledge; without spiritual difficulties, without any difficulties of faith, where would your faith be?—worthless.

We are not beasts, we do not walk by sight alone. We are men! We walk by faith, and reason, and sight. And when we say we have difficulties, we know that if we pursue them in all seriousness and in all sincerity, these difficulties are as nothing compared to the sureties of faith which we ought to follow. There is one more victory—I do not know why we call it so—but one very great victory which we have achieved in these later days, and the victory is this. I am perfectly justified in saying that at the present day there is no man of competent powers of observation and reflection who dares to stand up in any assembly and say that he is an atheist. This has become extinguished amongst all thoughtful men, and what the scientific man says now is not that there is no God—no, men of competent powers combine to say, "Yes, we do not know anything about God, but we will admit there is a great power behind the universe." But, oh, this is a splendid confession, this is being very near to the kingdom of heaven, for any serious man cannot go the length of acknowledging the existence of a supreme power behind the universe but in process of time—be it short or be it long—he will go a little further, and will be compelled to say, "I acknowledge this Being, and in no impersonal form. This power is no impersonal force; it is the eternal and loving Father of us all.'

